# Aberrant methylation of placental development genes in chorionic villi of spontaneous abortions with trisomy 16

**DOI:** 10.18699/vjgb-24-24

**Published:** 2024-04

**Authors:** O.Yu. Vasilyeva, E.N. Tolmacheva, A.E. Dmitriev, Ya.A. Darkova, E.A. Sazhenova, T.V. Nikitina, I.N. Lebedev, S.A. Vasilyev

**Affiliations:** Research Institute of Medical Genetics of the Tomsk National Research Medical Center of the Russian Academy of Sciences, Tomsk, Russia; Research Institute of Medical Genetics of the Tomsk National Research Medical Center of the Russian Academy of Sciences, Tomsk, Russia; National Research Tomsk State University, Tomsk, Russia; National Research Tomsk State University, Tomsk, Russia; Research Institute of Medical Genetics of the Tomsk National Research Medical Center of the Russian Academy of Sciences, Tomsk, Russia; Research Institute of Medical Genetics of the Tomsk National Research Medical Center of the Russian Academy of Sciences, Tomsk, Russia; Research Institute of Medical Genetics of the Tomsk National Research Medical Center of the Russian Academy of Sciences, Tomsk, Russia; Research Institute of Medical Genetics of the Tomsk National Research Medical Center of the Russian Academy of Sciences, Tomsk, Russia

**Keywords:** aneuploidy, trisomy 16, DNA methylation, chorionic villi, miscarriage, bisulfite sequencing, spontaneous abortions, анеуплоидия, трисомия 16, метилирование ДНК, ворсины хориона, невынашивание беременности, бисульфитное секвенирование, спонтанные абортусы

## Abstract

In humans, aneuploidy is incompatible with the birth of healthy children and mainly leads to the death of embryos in the early stages of development in the first trimester of pregnancy. Trisomy 16 is the most common aneuploidy among spontaneous abortions of the first trimester of pregnancy. However, the mechanisms leading to the death of embryos with trisomy 16 remain insufficiently investigated. One of these potential mechanisms is abnormal placental development, including aberrant remodeling of spiral arteries. Spiral artery remodeling involves the migration of trophoblast cells into the maternal spiral arteries, replacing their endothelium and remodeling to ensure a stable embryonic nutrition and oxygen supply. This is a complex process which depends on many factors from both the embryo and the mother. We analyzed the methylation level of seven genes (ADORA2B, NPR3, PRDM1, PSG2, PHTLH, SV2C, and TICAM2) involved in placental development in the chorionic villi of spontaneous abortions with trisomy 16 (n = 14), compared with spontaneous abortions with a normal karyotype (n = 31) and the control group of induced abortions (n = 10). To obtain sequencing libraries, targeted amplification of individual gene regions using designed oligonucleotide primers for bisulfite-converted DNA was used. The analysis was carried out using targeted bisulfite massive parallel sequencing. In the group of spontaneous abortions with trisomy 16, the level of methylation of the PRDM1 and PSG2 genes was significantly increased compared to induced abortions (p = 0.0004 and p = 0.0015, respectively). In the group of spontaneous abortions, there was no increase in the level of methylation of the PRDM1 and PSG2 genes, but the level of methylation of the ADORA2B gene was significantly increased compared to the induced abortions (p = 0.032). The results obtained indicate the potential mechanisms of the pathogenetic effect of trisomy 16 on the placental development with the participation of the studied genes

## Abstract (ru)

У человека анеуплоидия не совместима с рождением здоровых детей и в основном приводит к гибели эмбрионов на ранних стадиях развития в первом триместре беременности. Наиболее частая анеуплоидия среди спонтанных абортусов первого триместра беременности – трисомия 16. Однако механизмы, приводящие к гибели эмбрионов с трисомией 16, остаются недостаточно исследованными. Одним из таких потенциальных механизмов является нарушение развития плаценты, в том числе ремоделирования спиральных артерий. Ремоделирование спиральных артерий заключается в миграции клеток трофобласта в спиральные артерии матери и замещении их эндотелия для обеспечения стабильного питания эмбриона и снабжения кислородом. Это комплексный процесс, зависящий от множества факторов как со стороны эмбриона, так и со стороны матери. Нами проведен анализ уровня метилирования семи генов (ADORA2B, NPR3, PRDM1, PSG2, PHTLH, SV2C и TICAM2), участвующих в развитии плаценты, в ворсинах хориона спонтанных абортусов с трисомией 16 (n = 14), по сравнению со спонтанными абортусами с нормальным кариотипом (n = 31) и контрольной группой медицинских абортусов (n = 10). Для получения библиотек для секвенирования использована таргетная амплификация отдельных регионов генов с помощью разработанных олигонуклеотидных праймеров на бисульфит-конвертированной ДНК. Анализ проводили с применением таргетного бисульфитного массового параллельного секвенирования. В группе спонтанных абортусов с трисомией 16 был значимо повышен уровень метилирования генов PRDM1 и PSG2 по сравнению с медицинскими абортусами (p = 0.0004 и p = 0.0015 соответственно). В группе спонтанных абортусов с нормальным кариотипом не обнаружено повышения уровня метилирования генов PRDM1 и PSG2, но был значимо повышен уровень метилирования гена ADORA2B по сравнению с медицинскими абортусами (p = 0.032). Полученные результаты указывают на потенциальные механизмы патогенетического действия трисомии 16 на развитие плаценты с участием изученных генов.

## Introduction

Spontaneous abortion (miscarriage) is the spontaneous death of an embryo or fetus before 20 weeks of gestation. In the vast majority of cases, pregnancy is terminated when the embryo has life-incompatible genetic abnormalities. Slightly more than 50 % of all clinically diagnosed miscarriages are caused by aneuploidy, which mainly occurs during spermatogenesis or oogenesis, or in the early stages of embryonic development, and the most common aneuploidy among spontaneous abortions of the first trimester is trisomy 16 (Nikitina et al., 2016). Most of the aneuploid embryos die at the implantation stage, and the next peak of embryonic mortality is observed around 8–9 weeks of pregnancy. However, the mechanisms leading to the death of embryos with aneuploidy remain poorly understood.

In the first trimester of pregnancy, the most important process occurs: remodeling of spiral arteries, which consists in the migration of trophoblast cells into the spiral arteries of the mother, replacement of their endothelium and remodeling to ensure stable nutrition of the embryo and oxygen supply (Red- Horse et al., 2004; Jauniaux et al., 2006). This is a complex process that depends on many factors from both the embryo and the mother. Our preliminary results show that spontaneous first trimester abortions with an aneuploid karyotype have large-scale methylation disorders of repetitive sequences (Vasilyev et al., 2021) and genes playing an important role in placental development (Tolmacheva et al., 2022).

In this work, we conducted a more detailed study of part of the genes, the methylation disorders of which were previously detected in spontaneous abortions with trisomy 16 (PRDM1, PTHLH) (Tolmacheva et al., 2022) and a normal karyotype (ADORA2B, NPR3, PSG2, SV2C, and TICAM2) (unpublished data).

The ADORA2B gene is associated with remodeling of spiral arteries, and its hypermethylation is associated with impaired placental development, fetal growth retardation and the development of preeclampsia (Jia et al., 2012; Yeung et al., 2016). The NPR3 gene is a receptor for natriuretic peptide A, which plays an important role in the remodeling of the spiral arteries of the uterine wall (Zhang et al., 2021). Deficiency of natriuretic peptide A impairs trophoblast invasion and remodeling of spiral arteries, which leads to a phenotype similar to preeclampsia. The PRDM1 gene is a key regulator of terminal differentiation of giant trophoblast cells that replace the endothelium of the spiral arteries of the mother (Maioli et al., 2004). The PSG2 gene encodes pregnancy-specific beta-1 glycoprotein 2, the expression of which is increased in the trophoblast, and its increased level is observed in circulating trophoblast cells with true placenta accreta (Grunblatt et al., 2004). The PTHLH gene encodes osteostatin (parathyroid hormone-related protein), which is a precursor to a signaling peptide that plays a role in the differentiation of giant mouse trophoblast cells (Sandor et al., 2017). For the SV2C and TICAM2 genes, expression disorders are known in other pregnancy pathologies potentially associated with abnormal placentation (McMaster et al., 2004). The expression of the SV2C gene increases in exosomes in the blood of the mother with gestational diabetes compared with the group with normal pregnancy (Fang et al., 2021). Hypomethylation and high expression of the TICAM2 gene are also associated with preeclampsia and premature birth (Mason et al., 2011; Lim et al., 2020)

The aim of this study was to analyze the aberrant methylation of the genes of placental development ADORA2B, NPR3, PRDM1, PSG2, PTHLH, SV2C, and TICAM2 among spontaneous abortions of the first trimester of pregnancy with trisomy 16.

## Materials and methods

The analysis was performed on chorionic villi of spontaneous abortions with trisomy 16 (n = 14, gestational age 8.7 ± 1.6 weeks), spontaneous abortions with a normal karyotype (n = 31, gestational age 10.0 ± 2.2 weeks) and induced abortions (n = 10, gestational age 8.3 ± 1.2 weeks). Samples from the bio-collection “Biobank of the population of North Eurasia” of the Research Institute of Medical Genetics of the Tomsk National Research Medical Center of the Russian Academy of Sciences were used. Tissue samples were stored at a temperature of –80 °C. Informed parental consent was obtained for all samples to use the biomaterial for biobanking and research. The study was approved by the Committee on Biomedical Ethics of the Research Institute of Medical Genetics of the Tomsk NRMC (09.11.2020/No. 7).

The karyotype was determined using conventional cytogenetic analysis on direct preparations of chorionic villi and in fibroblast cultures of extraembryonic mesoderm (Lebedev et al., 2004). The presence of trisomy 16 was verified by fluorescent in situ hybridization (FISH) using subtelomeric DNA probes (16q and 16p) according to the described technique (Vasilyev et al., 2010).

Tissue separation was carried out morphologically, then chorionic villi cells were incubated overnight at 37 °C with proteinase K. The standard phenol-chloroform method was used to isolate DNA. Bisulfite DNA modification was performed using the EZ DNA Methylation-Direct kit (Zymo Research, USA) according to the manufacturer’s protocol. During bisulfite conversion, unmethylated cytosine is modified into uracil, which is replaced by thymine during further PCR, and methylated cytosine is not modified.

The methylation profile was analyzed using targeted bisulfite massive parallel sequencing. To obtain the libraries, the designed oligonucleotide primers were used to amplify the target regions of the ADORA2B, NPR3, PRDM1, PSG2, PTHLH, SV2C, and TICAM2 genes from bisulfite-converted DNA (see the Table). The choice of genes and target sites in them was determined by the differentially methylated CpG sites in chorionic villi of spontaneous abortions according to our preliminary results obtained using a large-scale methylation analysis (Tolmacheva et al., 2022), and their participation in the development of the placenta

**Table 1.**
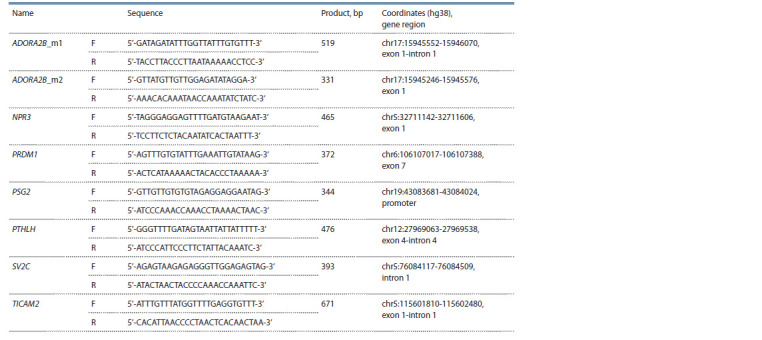
The average level of methylation of CpG sites in the target regions of the ADORA2B, NPR3, PRDM1, PSG2, PTHLH, SV2C, and TICAM2 genes in chorionic villi of spontaneous abortions with trisomy 16 compared with spontaneous abortions with a normal karyotype and induced abortions IA – induced abortions; SA NK – spontaneous abortions with a normal karyotype; SA Tri16 – spontaneous abortions with trisomy 16. The boxes represent the 25th and 75th percentiles, and the whiskers mark the minimum and maximum values. The square in the center of the box indicates the median, with blue lines marking the minimum and maximum values in the IA group. Above the figures, the proportion and number of spontaneous abortions with trisomy 16 with methylation levels of target genes beyond the limits of variation in the group of induced abortions are indicated (lowered – blue, elevated – red).

Amplification of target fragments was carried out using a set of HS-Taq PCR mastermix (2×) (Biolabmix, Russia) according to the manufacturer’s protocol with the following PCR conditions: 95 °C for 5 min; 36 cycles: 95 °C for 30 s, 60 °C for 45 s, 72 °C for 45 s. The concentration of the target fragments was determined using a Qubit 4.0 fluorimeter (Thermo Fisher Scientific, USA). The reaction products were purified using Sephadex G50 solution (Sigma, USA).

Targeted bisulfite massive parallel sequencing was performed on a MiSeq device (Illumina, USA) using a Micro kit (2x150). The quality of the reads was evaluated using FastQC v0.11.8, after which the remaining adapter sequences and low-quality reads were trimmed using Trim-Galore. The reads were then mapped to bisulfite-converted target sequences using the bwa-meth tool (v0.2.2) with default parameters. Methylation data in the context of CpG were extracted from the resulting BAM files using the MethylDackel tool. The results were presented as the methylation level, which is the ratio of the number of cytosines to the total number of cytosines and thymines in a individual CpG site. In addition, the average methylation level was calculated along all target sites. The statistical analysis was performed using the Statistica 10.0 software package (StatSoft, USA). The Mann–Whitney rank test was used to compare the methylation level between groups of samples. The differences were considered statistically significant at p < 0.05.

The study was conducted using the equipment of the center for collective use “Medical Genomics” of the Tomsk National Research Medical Center of the Russian Academy of Sciences

## Results

Significant differences in spontaneous abortions with trisomy 16 compared with induced abortions were observed in the following genes: PRDM1 (81.9 ± 2.8 % vs. 76.5 ± 2.6 %, p = 0.0004), PSG2 (51.6 ± 4.4 % vs. 44.6 ± 3.6 %, p = 0.001), and TICAM2 (4.5 ± 3.6 % vs. 12.5 ± 11.0 %, p = 0.044) (see the Figure). At the same time, the level of methylation of the PRDM1 and PSG2 genes in the group of spontaneous abortions with trisomy 16 was higher, and that of the TICAM2 gene was lower compared with induced abortions. In the group of spontaneous abortions with a normal karyotype, the level of methylation of the ADORA2B gene (m1 region) was significantly higher compared with the group of induced abortions (48.8 ± 15.3 % vs. 38.7 ± 10.2 %, p = 0.032) (see the Figure).

**Fig. 1.**
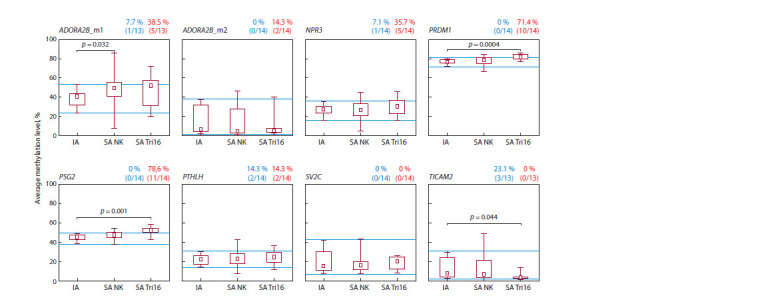
The average level of methylation of CpG sites in the target regions of the ADORA2B, NPR3, PRDM1, PSG2, PTHLH, SV2C, and TICAM2 genes in chorionic villi of spontaneous abortions with trisomy 16 compared with spontaneous abortions with a normal karyotype and induced abortions IA – induced abortions; SA NK – spontaneous abortions with a normal karyotype; SA Tri16 – spontaneous abortions with trisomy 16. The boxes represent the 25th and 75th percentiles, and the whiskers mark the minimum and maximum values. The square in the center of the box indicates the median, with blue lines marking the minimum and maximum values in the IA group. Above the figures, the proportion and number of spontaneous abortions with trisomy 16 with methylation levels of target genes beyond the limits of variation in the group of induced abortions are indicated (lowered – blue, elevated – red).

Some spontaneous abortions had methylation levels beyond the normal variability in the control group of induced abortions (see the Figure). The maximum number of spontaneous abortions with trisomy 16 with an increased level of methylation was found for the PSG2 gene (11 samples out of 14, which is 78.6 % of the total number of the studied samples) and for the PRDM1 gene (10 samples out of 14, 71.4 %) (see the Figure). Also, an increased level of methylation was observed in some spontaneous abortions with trisomy 16 for the following genes: ADORA2B_m1 (38.5 %), ADORA2B_m2 (14.3 %), NPR3 (35.7 %), and PTHLH (13.3 %). Lowered methylation levels in some spontaneous abortions were recorded only for the ADORA2B_m1 (7.7 %); NPR3 (7.1 %); PTHLH (14.3 %), and TICAM2 (23.1 %) genes. No spontaneous abortions with impaired methylation levels were detected for the SV2C gene (see the Figure).

## Discussion

Previously, our group and others showed methylation disorders in chorionic villi of spontaneous abortions with trisomy 16: increased methylation of retrotransposon LINE-1 (Vasilyev et al., 2021) and large-scale methylation disorders throughout the genome (Blair et al., 2014; Tolmacheva et al., 2022). In addition, methylation disorders in trisomy 16 were found to overlap with those in early-onset preeclampsia (Blair et al., 2014). Considering that one of the mechanisms of the development of preeclampsia is considered to be impaired placentation and remodeling of spiral arteries (McMaster et al., 2004), a possible mechanism leading to the death of embryos with trisomy 16 is abnormal methylation of placental development genes.

In this work, it was shown that in the studied group of spontaneous abortions with trisomy 16, the level of methylation of the PRDM1 and PSG2 genes was significantly increased compared with induced abortions. At the same time, the level of methylation of these genes did not significantly increase in the group of spontaneous abortions with a normal karyotype. Since the PRDM1 gene is a transcription factor, its effect is observed in many processes in the body. Recently, it was found that hypomethylation of the PRDM1 gene and a corresponding increase in gene expression in chorionic villi is associated with recurrent miscarriage (Du et al., 2020). Potentially, the negative effect of hypomethylation of PRDM1 may be associated with abnormal trophoblast migration and an increased level of trophoblast cell apoptosis (Du et al., 2020), as well as with the role of PRDM1 in regulating the transcription factor GATA2, which is key for trophoblast development (Paul et al., 2017).

It is possible that the increased methylation of the PRDM1 and PSG2 genes in the group of spontaneous abortions with trisomy 16 is associated with the effect of a supernumerary chromosome on the DNA methylation profile (Tolmacheva et al., 2013). This process is probably triggered by specific genes located on chromosome 16, which may be involved in the regulation of DNA methylation (Tolmacheva et al., 2022). In the context of this work, it is interesting that CTCF, one of the key regulators of chromatin conformation located on chromosome 16, can regulate the transcription of human PSG genes (PSG1–PSG9, PSG11) in trophoblast cells. Suppression of CTGF expression increased or suppressed the expression of several PSG genes, and this effect was accompanied by epigenetic changes (Jeong et al., 2021).

An interesting result requiring further study is a significant increase in the level of methylation of the ADORA2B gene in spontaneous abortions with a normal karyotype. It is likely that in individual embryos with a normal karyotype, methylation disorders of some genes involved in the development of the placenta may be caused by other causes unrelated to the influence of aneuploidy.

## Conclusion

The results indicate that the aberrant level of methylation of placental development genes may be an important factor associated with the death of embryos with trisomy 16 in the first trimester of pregnancy.

## Conflict of interest

The authors declare no conflict of interest.
